# Pharmaceutical subsidy policy in Iran: a qualitative stakeholder analysis

**DOI:** 10.1186/s12961-021-00762-6

**Published:** 2021-12-23

**Authors:** Alireza Olyaaeemanesh, Ebrahim Jaafaripooyan, Akbar Abdollahiasl, Majid Davari, Seyyed Meysam Mousavi, Mansoor Delpasand

**Affiliations:** 1 Health Equity Research and National Institute for Health Research, Tehran University of Medical Sciences, Tehran, Iran; 2 Department of Health Management and Economics, School of Public Health, Tehran University of Medical Sciences, Tehran, Iran; 3 Pharmacoeconomics and Pharmaceutical Administration, Tehran University of Medical Sciences, Tehran, Iran; 4 Management and Leadership in Medical Education Research Center, Kerman University of Medical Sciences, Kerman, Iran; 5 Health Foresight and Innovation Research Center, Health Services Management Research Center, Institute for Futures Studies in Health, Kerman University of Medical Sciences, Kerman, Iran; 6 Iranian Social security organization, Tehran, Iran

**Keywords:** Pharmaceutical subsidy, Stakeholder analysis, Iran

## Abstract

**Background:**

Over the past three decades, allocation of foreign currency subsidies has been the primary strategy of various administrations in Iran to improve access to medicines. This strategy has resulted in several challenges, including stakeholder conflicts of interest.

**Objective:**

To identify the power, interest, and role of the stakeholders in allocating foreign currency subsidies to medicines in the Iranian health system.

**Methods:**

In this qualitative study, 39 semi-structured interviews were conducted. Key informants were recruited using a purposive sampling technique. The theoretical framework adopted by Varvasovszky and Brugha was employed. The data were analysed using directed content analysis.

**Results:**

The foreign currency subsidy for medicines included 21 stakeholders in five main categories: governmental organizations, Iranian Parliament, general population, nongovernmental organizations (NGOs), and the pharmaceutical industry. Stakeholders varied in their level of participation and support in the policy-making process. Among them, the Iranian Government, Planning and Budget Organization, the Ministry of Health and Medical Education (MoHME), and Iran Food and Drug Administration (IFDA) were the most important stakeholders, with highly supportive positions, while domestic drug manufacturers were the strongest opponents of this policy. The Government of Iran is the most powerful institution with regard to the ability to allocate foreign currency subsidies to medicines, followed by the MoHME and the IFDA.

**Conclusion:**

This study demonstrated that identifying and analysing the stakeholders involved in allocating foreign currency subsidies to medicines can provide valuable information for policy-makers to enable a more comprehensive understanding and better capacity to determine whether or not to eliminate these subsidies. Moreover, decision-making in this process is a long-term issue that requires consensus among all stakeholders. Because of the political and social consequences of eliminating foreign currency subsidies, the necessary political will is not institutionalized. We recommend a step-by-step approach in eliminating foreign currency subsidies if the requirements are met (i.e., those related to the consequences of such interventions). Therefore, revision of the current policy along with these requirements, in addition to financial transparency and enhanced efficiency, will facilitate progress towards achieving the Sustainable Development Goals by improving access to medicines.

**Supplementary Information:**

The online version contains supplementary material available at 10.1186/s12961-021-00762-6.

## Background

Target 8e of the Millennium Development Goals (MDGs) emphasizes ensuring access to affordable essential medicines in developing countries in cooperation with pharmaceutical companies [1]. Regarding the assessment of progress towards achieving this goal, the United Nations (UN) report indicates that high-cost and unaffordable medicines are a major barrier to access to treatment in low- and middle-income countries (LMICs) [2, 3]. Thus, in 2015, 193 UN Member States adopted the Sustainable Development Goals (SDGs) [4]. Target 8 of SDG 3 (Target 3.8) includes the achievement of universal health coverage (UHC), defined as financial risk protection, access to quality essential health-care services and access to safe, effective, quality, and affordable essential medicines and vaccines for all [5]. WHO defines UHC as ensuring that everyone who needs care has adequate access to quality health services, including promotional, preventive, curative, rehabilitative, and palliative care services, without exposure to financial suffering. Therefore, UHC captures two interrelated dimensions: service coverage (SDG indicator 3.8.1) and financial protection (SDG indicator 3.8.2) [6].

In many parts of the world, out-of-pocket (OOP) expenditure is still high. Drug costs are often mentioned as the first or second cause of OOP payments which can be catastrophic, shifting people from the middle and lower social classes into destitution in the event of a severe or chronic illness [7]. A study among 51 different countries showed that the cost of medicine, compared to that of hospitalization and outpatient services, causes more families to suffer financial hardship [8]. According to the World Bank, 20–50% of total health expenditures (THEs) in developing countries are related to pharmaceutical expenditures [9]. A survey of 51 countries found that the poor spend about 41% of their THEs for medicines [10]. Since most health expenditures are associated with medicines, improving access to quality essential medicines is critical to achieving UHC and progress towards the SDGs [11, 12].

The Islamic Republic of Iran, located in the Middle East, is deemed a developing country but is situated economically among the upper-middle-income countries. With a population of over than 83 million, Iran was ranked as the world’s 18th largest country in 2020 [13]. Its gross domestic product (GDP) has been estimated at US$ 628 billion for the Iranian calendar year 2020–21 [14]. The Iranian health system has a well-defined three-tier structure: primary, secondary, and tertiary [15]. Healthcare services are financed through multiple sources, including government funds, general taxation, health insurance, individual donations, and OOP payments. It is noteworthy that there is fragmentation in insurance funds [16]. Despite legislation establishing a single national insurance scheme in 2010, physical integration of health insurance funds was never implemented [17]. In recent years, Iran has spent about 9% of GDP on health (equivalent to US$ 475 per capita), of which approximately 1.4% (equivalent to US$ 75 per capita) was allocated to the pharmaceutical sector [18, 19].

Iran has a generic-based pharma market [20]. For instance, the numbers of Iranian pharmaceutical manufacturing, import, and distribution companies were 185, 232, and 50, respectively, in 2018 [21]. Despite the fact that the proportion of marketed pharmaceuticals produced locally in Iran has reached more than 95% (in terms of sales volume) according to the national drug policy (NDP) [21, 22], the local manufacturer share of the pharma market value is still low because of the dependence on the importation of raw materials for the production of these medicines. According to a study, more than half of the active pharmaceutical ingredients (API) are produced in the country, and the remaining are supplied by reputable companies in India and China, and in some cases by some European companies [23]. According to the previous statistics, domestically produced drugs account for more than 70% of Iran's pharmaceutical market value, but a recent report by the Research Center of the Islamic Parliament mentioned a value of 23% [24].

In 2018, the Iran Food and Drug Administration (IFDA) reported that the funds allocated to API and end products was about US$ 1 million (42%) and US$ 1.4 million (58%), respectively [21]. Although efforts have been made in recent years to reduce the amount of currency allocated to medicines, all such efforts have demonstrated Iran’s dependence on foreign countries in the field of pharmaceutical manufacture [25]. For example, one analysis of data from the Central Bank of Iran (CBI), published by Iran Open Data, shows that approximately US$ 43.8 billion in government-subsidized funds were earmarked for the import of essential goods, with nearly US$ 4.2 billion allocated to pharmaceutical companies as a preferential currency (42,000 Iranian rial) in the 2 years leading up to 2020 [26].

Also, according to the national budget law of 2018, about 100,000 billion Iranian rial (equivalent to US$ 2.4 billion of foreign currency) in subsidies has been allocated to medicines [27]. However, a report published by the Planning and Budget Organization (PBO) in 2018 challenged this figure [28], and indicated that only 2% of this subsidy reached those in the first decile, while the tenth decile, the most affluent one, had received about 39% of these resources. This report also showed that households in the first decile have only received 1–6% of subsidies allocated to medicines, while this number for the last three deciles ranges from 15 to 39%.

Other reasons for the low share of local manufacturing within the pharma market include the chemical and biological products that are not produced locally due to their high-tech nature, such as new monoclonal antibodies, novel anticancer agents, and new recombinant proteins [22]. These medicines are very expensive and were not included in the basic health insurance benefit packages (BHIBPs), or access to them was impeded by high copayments. Despite the availability of insurance coverage, there is remarkable inequity in terms of access to healthcare services due to the variation in coverage, as well as amounts of coinsurance for the same services [29]. As a result, according to the National Health Accounts [30] and a study in 2019 [17], OOP expenses for the whole population escalated from 46.2% (in 2003) to 53.8% of THEs in 2008, and then dropped to 38.8% in 2016. Drug costs and expenses associated with drug consumption in Iran, similar to many developing countries, are about 30% of the THEs and approximately 50% of outpatient healthcare expenses [31]. Hence, considering the government's role in ensuring access to medicine, in 2008 more than US$ 300 million was allocated to cover the drug subsidies by the Ministry of Health and Medical Education (MoHME), and this amount in recent years has increased to approximately US$ 400 million. Medicines for haemophilia, thalassemia, transplant, and multiple sclerosis (MS) patients are the major items that are subsidized by the MoHME [21, 22]. Imported medicines for treatment of cancers, MS, and nerve diseases account for almost 5% of the total medicine imports [32].

The IFDA was established with the approval of the Supreme Administrative Council in 2010 [21]. Pricing of pharmaceuticals in Iran is highly regulated, and all prices are set by the IFDA [32]. During the past decade, Iran has experienced several periods of extreme exchange rate volatility, which had lasting implications for the economy and health [33, 34]. Hence, in accordance with upstream laws, including the 5-year national development plans (NDPs), policy-makers have undertaken huge efforts to allocate subsidies for medicines and essential goods to deal with the effects of exchange rate changes and fluctuations.

During economic fluctuations, the gap between the official exchange rate and its market price widened, and the hidden subsidy of medicines experienced greater challenges. According to previous studies, sanctions imposed in 2012 by the United States against Iran and the consequent economic downturn, such as the decline of local currency, have increased the price of medicines, especially those for special, incurable, and chronic diseases. Consequently, the access to essential medicines for 6 million patients with haemophilia or thalassemia, patients with chronic kidney disease on dialysis, those who received transplantation, and those with MS or cancer has been compromised [35–38].

At least three different exchange rates are utilized as a reference for foreign currency-denominated transactions. The first rate is the official one, which is constant (42,000 Iranian rial per 1 US dollar) and dependent on subsidizing the importation of essential goods. The second is the Forex Management Integrated System (NIMA) rate, a quasi-market rate. The NIMA rate is managed by the CBI, in which main exporters encompassing the petrochemical sector producers are needed to market their export proceeds. Eventually, other minor demands such as travel and cash are met through a parallel market by banks and money exchange shops for limited amounts [39].

Making decisions on pharmaceutical subsidies is always a substantial obstacle for policy-makers in the Iranian healthcare system due to the differences in context between the Iranian pharmaceutical system and that in other developing countries. Making such decisions on pharmaceutical subsidy in Iran historically reflects the nature of the healthcare system during the last three decades, and this might be due to the imposed sanctions, and fluctuations in the exchange rate which dramatically decrease the value of the local currency. This is deemed a special issue of which Iran suffers differently from other developing countries.

Access to medications depends on availability, affordability, quality, and appropriate use, and involves a complex interaction between governments, pharmaceutical firms, and general population [40–42]. Financial protection policies against pharmaceutical expenditures are also of special attention in the sophisticated and long-term plans in Iran, including the 5-year development plans and annual budgets. As an essential document in this field, the National Pharmaceutical Policy of the Islamic Republic of Iran has categorized the pharmaceutical system's general policies and orientations into 13 principles. Concerning accessibility, the MoHME is explicitly obliged to provide patients with the necessary, affordable, and high-quality medicines in a timely manner [43]. Therefore, medicine, as a strategic commodity subject to various subsidies, is a basic and fundamental public need, with a complex and multifaceted nature that requires a national effort and the involvement of all stakeholders [44].

Health-related issues are complex and multifactorial, and various stakeholders involved in the policy-making process may directly or indirectly influence, promote, or even undermine the impact of policies [45, 46]. Stakeholders include all individuals, groups, and organizations that can influence a health-related issue, and have diverse views that might affect the process of policy implementation [47]. Understanding and identifying stakeholders can increase awareness about different aspects of the decision-making process and potentially lead to its improvement. Stakeholder analysis is a systematic process of identifying stakeholders in terms of interests, power, and relationships with a health-related policy [47].

In addition to the scarce resources and the various priorities in the health system, health policy-makers are faced with making difficult decisions regarding the financial protection of the population against catastrophic health expenditures, especially those people who experience special, incurable, and chronic diseases. A review of high-level documents developed during the past few decades revealed that the Islamic Republic of Iran had adopted the policy of allocating foreign currency subsidy (hidden subsidy) to protect people against intolerable health expenditures. Decisions regarding the allocation of subsidies for a drug are made through a complicated process involving several actors and institutions in a specific social, political, and economic context. This process rarely considers the interests and priorities of involved stakeholders, including the MoHME, health insurance funds, pharmaceutical firms, and nongovernmental organizations (NGOs). Decisions regarding foreign currency subsidies are not made based on scientific evidence; rather, they are heavily influenced by individuals’ and organizations' interests, values, and power. Accordingly, to achieve a transparent and evidence-based approach for decision-making, it is necessary to thoroughly evaluate and investigate various factors that affect the adoption and implementation of the foreign currency subsidy policy. In this regard, stakeholder analysis is a key tool for assessing multiple issues [48–50].

## Objective

To analyse the stakeholders involved in allocating foreign currency subsidies to medicines in the Iranian health system.

## Methods

In this qualitative study, stakeholder analysis was used as the primary method of data analysis. This method is defined as the systematic process of collecting and analysing qualitative information to determine the interests of individuals when developing or implementing policies or programs [51]. Based on our objective, the theoretical framework adopted by Varvasovszky and Brugha [45] was used to investigate the different dimensions and effects of stakeholders on the policy of foreign currency subsidy for medicines in Iran.

The analysis used in this study includes identifying the main stakeholders and their perspectives regarding the present policy of foreign currency subsidy for medicines, their power and authority to take action, and their positions on continuation of the policy. The stakeholders include a set of governmental organizations and NGOs that potentially influence the policy-making process differently based on their interests and power. Based on the inductive interpretation of interviews, stakeholders, according to their interest and power, have been classified into those with high or low impact on subsidy policy for medicines and putting the subject on the agenda. Interest was identified as the extent of involvement in the policy. The position was defined as the interaction with other actors involved in the policy. Power was defined as the degree or extent to which stakeholders are likely to influence the policy. We aimed to invite various stakeholders involved in the policy to ensure a diversity of viewpoints.

A purposive sampling technique was applied [52–54]. Initially, the research team convened a meeting to determine the details of the sampling to recruit key informants. Considering that the main purpose of the research was to analyse the stakeholders of subsidy policy, a preliminary list of stakeholders and actors related to the medicine subsidy policy was first compiled to achieve maximum diversity. The experience of the research team members facilitated the identification of the initial list of actors and stakeholders. It should be noted that two members of the research team have participated in previous meetings related to decisions on the provision and allocation of financial resources for medicine. Then, based on research team members’ knowledge and experience regarding the research context in each group of stakeholders identified in the initial list, the key informants to be invited for an interview were identified. After interviewing the individuals identified in the initial list, the interviewees also introduced a number of key informants and added them to the same list. Criteria for selecting the initial list included familiarity with health issues, especially allocation of financial resources to medicine, and work experience of not less than three years. In this study, we tried to sample a wide array of perspectives, including parliament members, experts of the PBO, the Secretariat of the Supreme Insurance Council, MoHME, medical universities, IFDA, health insurance organizations, the Transparency and Justice Watch group, the pharmaceutical industry, and the Charity Foundation for Special Diseases (CFSD), to avoid bias in selecting interviewees. Before the interviews were conducted, the objectives of the study were explained by phone calls or face-to-face conversations.

As illustrated in Table 1, 39 semi-structured interviews were performed by one of the research team (MD) based on an interview guide which was developed through internal discussion. The interview guide included interviewees' characteristics and items related to the study objectives (Additional file 1). In addition to audio recording, field notes were taken for greater accuracy in data collection. The interviews lasted 75 minutes on average, and were held in the interviewees’ offices between 2017 and 2019. After transcribing the interviews, confidentiality was preserved by removal of the personal information.

**Table 1:** List of the participating individuals and organizations in the present study

Organizational affiliation	Description	Number	Total
Iranian Parliament (health commission)	Physician (Ear, Nose, and Throat specialist)	1	2
	Physician (Ophthalmologist)	1	
Planning and Budget Organization	Physician (PhD in health policy)	1	3
	Physician (PhD in Physiology)	1	
	Expert	1	
Ministry of Health and Medical Education	Physician (Radiotherapist)	1	4
	Physician (PhD in Nutrition)	1	
	Physician (PhD in Social Medicine)	1	
	Physician	1	
Ministry of Cooperatives, Labour, and Social Welfare	Pharmacist	1	1
Iran Food and Drug Administration	Pharmacist (PhD in Pharmaceutics)	1	5
	Pharmacist (PhD in Pharmaceutical Economics and Management)	3	
	Pharmacist (PhD in Pharmacology)	1	
Pharmaceutical industry (manufacturers, importers, and distributors of medicines)	Pharmacist	2	5
	Chemical Engineering	1	
	Pharmacist (PhD in Pharmacology)	1	
	Pharmacist (PhD in Physiology)	1	
Health insurance organizations			
Iran Health Insurance Organization	Physician (Ophthalmologist	1	3
	Physician (Ph.D. in Epidemiology)	1	
	Physician (Ph.D. in Health Policy)	1	
Social Security Organization	Pharmacist	1	3
	Physician	1	
	Physician (Ph.D. in Health Economist)	1	
Imam Khomeini Relief Foundation (merged with the Iran Health Insurance Organization)	Physician	1	2
	Nurse	1	
Armed Forces Medical Services Insurance Organization	Physician	1	1
Medical universities	Physician (Ph.D. in Pharmacology)	1	4
	Pharmacist (Ph.D. in Pharmacology)	1	
	Pharmacist (Ph.D. in Health Economist)	1	
	Pharmacist (Ph.D. in Pharmaceutical Economics and Management)	1	
Transparency and Justice Watch	Expert	1	1
Support associations	Physician (neurologist and MS fellowship)	1	3
	Pharmacist	1	
	Law expert	1	
Cancer Research Center	Physician (cancer surgeon)	1	1
Tehran Chamber of Commerce	Pharmacist	1	1
Total	39		

### Data analysis

We analysed the interview data using a directed content analysis (DCA) approach [55, 56]. This approach is appropriate when theory or prior research exist about a phenomenon. Hence, in the first phase, all transcripts were carefully read and checked for accuracy by comparing them with the audio recordings. In the next phase, and by applying MAXQDA 2020 (VERBI Software, 2019), one member of the research team (MD), in discussion with AO, drew up a primary coding framework guided by the research questions. The primary coding framework was then provided to the research team to enable maximum chance of reflexivity and the opportunity to explore and resolve uncertainties. In regular discussion among the research team, the codes were then aggregated or grouped together into main overarching categories of a predefined stakeholder analysis framework. Eventually, the set of categories and selected quotes for each stakeholder were discussed and agreed upon.

In the next phase, the role of each stakeholder was described according to the analysis of transcripts. Power and interest were grouped into three categories: low, medium, and high. The position was classified into five categories: low, moderate, high, neutral, and opposing. The classification of stakeholder roles was performed by a member of the research team (MD) and was then examined and approved by the research team.

### Rigor

Various strategies have been introduced to increase trustworthiness in qualitative studies. According to Lincoln and Guba’s approach [57], we used four criteria including credibility, dependability, confirmability, and transferability. To ensure that our findings were true, credible, and believable, the interviews were performed by an experienced and highly skilled researcher who was very familiar with the Iranian health system and practically engaged in the problems of patient access to these medicines, especially the expensive medicines used for cancer, thalassemia, and diabetes. In addition, the interview guide, as our data collection protocol, was piloted with several interviewees. This study applied semi-structured interviews and the completion of member checks to ensure the accuracy of the research results. We also used peer debriefing, which entailed the qualitative lead researcher (MD) seeking support from the senior researcher (AO) to provide scholarly guidance.

We also collected rich and detailed descriptive information about the key informants to increase transferability. However, to ensure and protect anonymity, all reporting data were anonymized to ensure that the description of participants and selected quotes were not attributable to participating key informants.

To verify the dependability of the data, researchers trained in interview skills developed a data collection and analysis process. The study protocol was also prepared and approved by the Committee for Research at the Tehran University of Medical Sciences (TUMS). Moreover, the researchers carefully recorded all interviews, and to ensure data coding accuracy, we proofread and checked interviews at regular meetings.

We ensured confirmability by documenting the justification of methodological and analytical choices to illustrate how the data were derived in relation to the study objectives, and transparently described the research steps taken from the start of the project to the development and reporting of the findings.

### Ethical considerations

Written informed consent was obtained from all participants before holding the interviews. The research protocol for this study was approved by the Ethical Review Committee of the Tehran University of Medical Sciences (code: IR.TUMS.SPH.REC.1395.1786).

## Results

The findings regarding foreign currency subsidy policy for medicines included stakeholders in five main categories: Iranian Parliament, governmental organizations, NGOs, general population﻿, and the pharmaceutical industry. The actors in the process included individuals and representatives from parliament; PBO; the Deputy for Treatment Affairs of the MoHME; the Ministry of Cooperatives, Labour, and Social Welfare (MCLSW); health insurance organizations; support associations; charity organizations; scientific associations; media; the CBI; the Ministry of Industry, Mining, and Trade (MIMT); the CFSD; the Customs Administration; and the pharmaceutical industry. Each actor's status in the policy-making process was determined in three dimensions: interest, position, and power (Table 2). The score for each dimension was determined based on the interviewees' statements about stakeholder status regarding the foreign currency subsidy policy for medicines.

**Table 2:** Interest, influence, and position of stakeholders

Actor/stakeholder	Interest	Influence/power	Position
General population	Low	Low	Neutral
Iranian Parliament	Moderate	High	Moderate support
Government	High	High	High support
Planning and Budget Organization	High	High	High support
Ministry of Health and Medical Education (MoHME)	Moderate	High	High support
Special Diseases Department in the MoHME	Low	Moderate	Low support
Ministry of Cooperatives, Labour, and Social Welfare	Low	Low	Neutral
Iran Food and Drug Administration	High	High	High support
Health insurance organizations			
Social Security Organization	Low	Moderate	High support
Iran Health Insurance Organization	Low	Moderate	Moderate support
Other Health Insurance Organizations	Low	Moderate	Neutral
Scientific associations	Low	Low	Neutral
Support associations	High	Moderate	Moderate support
Charity Foundation for Special Diseases	High	Moderate	Moderate support
Media	Low	High	Neutral
Central Bank of Iran	Moderate	High	Moderate support
Ministry of Industry, Mining and Trade	Low	Moderate	Low support
Pharmaceutical industry			
Manufacturers	Moderate	Moderate	Opposition
Importers	High	Moderate	Moderate support
Distributors and exporters	Low	Low	Neutral
Customs Administration	Low	Low	Low support

*IFDA:* According to the Medical, Pharmaceutical, Food, and Beverage Regulation Act, approved in 1955 and modified in 1988, the IFDA is the steward charged with supplying and monitoring medicines in the country. The Deputy for Food and Medicine was merged with the Deputy for Treatment Affairs of the MoHME, following the policy of merging parallel institutions. At the end of 1997, the Deputy for Food and Drug was established once again, and in 2010, the IFDA was also established. Therefore, the revival of the food and drug deputy position, and then its transformation into an independent organization as the leading institution for monitoring the food and drug sector, gave it authority over the pharmaceutical sector. The IFDA is highly interested in the foreign currency subsidy policy for medicines, which is rooted in its mission and philosophy; according to the law, the IFDA is responsible for supplying quality medicines at reasonable prices. In this regard, the IFDA has argued that elimination of foreign currency subsidies for medicines would adversely affect the accessibility of medicines; therefore, the IFDA opposed this proposal, which is somewhat in line with the MoHME policy.

Interviewee P1 noted that, according to the abovementioned laws and the Establishment law of MoHME (1988), the IFDA has a wide set of responsibilities and authority, ranging from monitoring the quality to pricing, licensing, and allocating foreign currency subsidies. Hence, some of its duties are in competition with each other, and this in turn might lead to making subjective and irrational decisions. Separation of duties and delegation of some tasks to other organizations can improve this situation, and help the IFDA better focus on its primary mission.

*General population:* General population are stakeholders who are directly affected by the policy of foreign currency subsidy for medicines. Thus, any change in the subsidy allocation procedure directly touches their lives through OOP payments. Several disease organizations, due to their power, influence the allocation of subsidies. The critical point is that, similar to some other countries, in Iran, general population are not involved in decision-making and do not have an organized power to influence the policy process. Although general population are not directly involved in decisions, some interviewees argued that they indirectly influence such decisions by electing the president and parliament members, and selecting the health benefit scheme.

*Health insurance funds represent the general population because our proposals to the IFDA for drug subsidies are also based on the public opinion.* (P2)

Another interviewee noted:

*General population have a secondary role, as they elect members of the parliament and the government.* (P3)

On the other hand, since 1979, a fully democratic system has been implemented in Iran, so people elect the president, and the president selects the cabinet. General population also elect the parliament members, and they in turn vote for the ministers as well. Therefore, general population's participation in decision-making is indirect but crucial, especially when we realize that people are the primary payers of subsidies. Furthermore, discussing the prevention of contact-related diseases such as contagious diseases, general population's opinions regarding access to medicines for such diseases become stronger and more apparent.

*After all, general population don't like to live with patients who suffer from leprosy or tuberculosis (TB). On the contrary, imagine that the MoHME has announced that since now they can freely live in society, which means infecting many healthy people. Here, the general population's role will be highlighted, as they are going to protest; particularly when a drug-resistant TB case has been identified, the health system should provide the patient with medicines to keep him isolated from society. This patient should not be allowed to use the public transportation system. * (P4)

*The Islamic Consultative Assembly (Iranian Parliament):* In recent years, the parliament has enacted important reforms, such as establishing the food and drug faction and considering epidermolysis bullosa (EB) as a special disease, which indicates its commitment to health-related affairs. In addition, the parliament is strongly committed to allocating foreign currency to medicines related to special diseases. The Health Commission is one of the parliament's specialized bodies that deals directly with the health system's affairs.

The Iranian Parliament also plays a key role in foreign currency subsidy policy for medicines, because all government measures must be approved by the parliament in terms of planning, legislation, and budget allocation; thus, it can be argued that the parliament has a supportive and reformist role. As the country's highest legislative body, the parliament also has the authority to hold the government accountable and even to mandate its compliance with the laws.

*Parliament has a supportive and reformist role and can intervene when the government raises the price of public services or medicines.* (P3)

Therefore, the parliament plays a significant role concerning the policy of foreign currency subsidy for medicines. Although some parliament members have recently opposed this policy, the majority are interested in the foreign currency subsidy policy for medicines and have supported it several times, and this constitutes a substantial power. By hearing the voice of media and support associations, the parliament plays a key role in the inclusion of certain diseases in the list of specific diseases receiving subsidies.

*The parliament can change the Targeted Subsidy Act. For example, increasing the monthly pensions by two times. It also has a key role in allocating subsidies to medicines.* (P5)

*Government:* The government is responsible for executing the law; thus, it is the strongest influence in determining the policy of foreign currency subsidy for medicines. Concerning the government's position, based on the interviews with stakeholders, it can be stated that the government is reluctant to reduce the subsidies due to the sensitive political and social consequences. Therefore, it seems that the political will is not present among politicians. Despite being aware of the challenges and problems of the foreign currency subsidy for medicines, the government holds a supportive position.

*PBO:* The PBO is aligned with the government and has great power and interest regarding the subsidy policy. Although the PBO is aware of the challenges and problems of this policy, it has a supportive position practically; however, one of the interviewees, who was a manager of the PBO, expressed his opposition:

*The PBO has an important role in allocating subsidies. Indeed, it has a key role. I think one of the central policies that we should have for the country's pharmaceutical system is that the pharmaceutical sector should not receive any foreign currency subsidy, whether domestic production or imported ones.* (P5)

*Deputy for Treatment Affairs of the MoHME:* The MoHME has a specialized department for special diseases that supervises the quality of treatments provided to patients with specific diseases using a set of indicators and standards, and it has a demanding position regarding the IFDA. By receiving indirect feedback about the effect of medications on treatment and quality of life of patients, and regular communication of this feedback with the IFDA, this actor has a major impact on foreign currency subsidy policy for medicines. Drawing on information about the number of patients needing a particular drug, and according to the protocols and guidelines, the Special Diseases Department officially reports the annual amount of required pharmaceutical items to the IFDA via the Deputy for Treatment Affairs in the MoHME. Therefore, this department has an advisory role to the IFDA regarding allocating foreign currency subsidies for medicines. Interviewee P6 noted that the mission and purpose of establishing this department in the MoHME had been supporting patients experiencing specific diseases. However, as the IFDA is an independent organization and is responsible for supplying medicines in the country, the Department of Special Diseases does not significantly impact allocation of subsidies to medicines, despite having official organizational power.

*MoHME:* This ministry is among the main actors involved in foreign currency subsidy policy for medicines. Its position is somewhat different with regard to the method of subsidy allocation. In this respect, as interviewee P7 noted, the MoHME opposes the elimination of subsidies and believes that producers and importers of pharmaceutical products cannot obtain foreign currency through the government's primary market, known as NIMA. Hence, the stewardship role of the MoHME and its supportive position against cancellation of foreign currency subsidies has to be considered. The MoHME would accept the elimination of this policy only when the political context is favourable and the foreign currency subsidy for other commodities is eliminated, and if the gap between official and market exchange rates is addressed by being paid to health insurance funds. Thus, this augments the supportive role of the MoHME towards this policy.

*MCLSW:* As the steward of health insurance organizations, the MCLSW is a chief actor in the foreign currency subsidy policy for medicines. Nevertheless, the MCLSW does not have a positive or negative position regarding this policy, nor has it announced any policy position. For that, in recent years, the Iran Health Insurance Organization (IHIO) has been transferred from the MCLSW to the MoHME, and this ministry is less interested in the policy of concern.

*MIMT:* This ministry is another stakeholder of foreign currency subsidy policy for medicines. The IFDA sends all requests for importing medicines to the MIMT via a specially developed portal known as Titak. Then, the MIMT evaluates the requests and informs the CBI for allocating foreign currency. An interviewee noted that the MIMT is only a supply department and has little interest in this issue:

*There is no complete coordination between various actors yet. The trading system (i.e., NIMA) is recently developed and still has room for improvement.* (P8)

In this regard, MIMT has moderate power and a moderate to low supportive position due to the lack of interest. This ministry is a stakeholder that can play a more prominent role in this field due to its nature and engagement in the business.

*Health insurance organizations:* There are four prominent health insurance organizations in the country, namely the Social Security Organization (SSO), IHIO, the Armed Forces Medical Services Insurance Organization (AFMSIO), and the Imam Khomeini Relief Foundation (IKRF). These organizations are engaged in designing the policy of foreign currency subsidy for medicines. The SSO is a nongovernmental and independent institution. The board of trustees of the organization appoints the chief executive officer of the SSO. For several years, the IHIO was affiliated with the MCLSW, but it is currently politically affiliated with the MoHME. The IKRF covers a small proportion of the population, and it was recently merged with the IHIO.

For national security reasons, the AFMSIO is an independent organization and has a limited role in the subsidy policy. Therefore, the IHIO and SSO play a decisive role in the policy of foreign currency subsidy for medicines, and other insurance funds are subject to these two organizations' decisions. The IHIO approves the persistence of the current policy, but it will follow the Ministry's position since it is politically affiliated with the MoHME. The SSO has a different situation, so that the SSO is against abandoning foreign currency subsidies due to the government's refusal to pay its previous debts to the organization.

*“It is about two years of debate on removing the foreign currency subsidy (which is around 4 billion dollars) to medicines and transferring the gap between market exchange rate and the subsidized rate (4200 Iranian rial) to health insurance organizations”.* (P9)

*“According to our experience, the government won't pay any money to us. The PBO, which is directly affiliated with the government, tries to cheat health insurance organizations. Then, they say, oh, all health insurance funds are bankrupted, what you have done?”* (P9)

*The pharmaceutical industry:* Despite the importance of the private sector, including manufacturers, importers, exporters, distribution organizations, and pharmacies, this sector does not officially play a significant role in foreign currency subsidy for medicines. However, private sector organizations such as pharmaceutical syndicates and health economics federations can also play an active role. This is subject to official acceptance of this sector by state actors. The primary goal of manufacturers, importers, and distributors engaged in the drug supply chain is to increase market volume by selling as much medicine as possible, increasing revenues. Increased subsidies mean lower prices and larger market size; however, importers and manufacturers gain a higher benefit than other supply chain actors. Therefore, the benefits of this group of stakeholders, particularly manufacturers and importers, strongly depend on allocating subsidies to medicines. As a result, these actors support foreign currency subsidy policy for medicines; however, they do not have great power to influence such policies and are dependent on pharmaceutical associations, including syndicates. Concerning the policy of foreign currency subsidy for medicines, it seems that all parts of the drug supply chain are against this policy; however, importers support this policy in practice, while manufacturers are opposed to the policy, whereas exporters have a “neutral” position.

The opposition among domestic manufacturers is attributed to the method of subsidy allocation.

*“For importers, currently, we are allocating subsidies to all materials of the production including the primary active ingredients, other materials, requirements, and packaging, for both final products and semi-final products. Only the primary ingredient is subsidized for producers, which composes 30 to 40% of the total price depending on the drug, but does not include other components such as packaging, salaries, and energy-related costs (fuel and electricity). This policy has resulted in an increased price of domestic products*”. (P5)

Interviewees P5, P10, and P11 noted that the country's current foreign currency subsidy allocation model is discriminative against domestic producers, which means privileging importers and somewhat weakening domestic production of medicines in the year so-called "supporting national production".

Interviewee P11 stated that this discrimination against domestic producers is rooted in ignoring those experienced in this field. The interviewee also emphasized that, concerning foreign currency subsidy policy for medicines, the decision-making system should take a bottom-up approach.

*CBI:* The CBI is an Iranian financial and banking monitoring institution established in 1960. According to Article 10 of Iran's monetary and banking law, the CBI is responsible for regulating and implementing credit and monetary policy. Regulating the foreign exchange rate of rial transactions and monitoring foreign currency issuance and entry are some of the CBI's primary responsibilities. Therefore, the CBI does not play an essential role in the pharmaceutical memoranda of understanding, but it has a significant role in allocating foreign currency to medicines. After submitting the foreign currency allocation priorities by the MIMT, the CBI coordinates with the destination bank for releasing the money; accordingly, the Customs Administration then receives the goods. The CBI is powerful in the policy of foreign currency subsidy for medicines; however, it does not show much interest and has a moderate-to-low supportive position owing to the increased workload.

*Customs Administration:* After delivering the drug through the Titak system, the customs administration issues the necessary administrative approvals. After review of the product's authenticity and labelling, the product will be shipped to the pharmaceutical warehouse and distribution companies. Hence, the customs administration has little power or interest and has a medium-to-low supportive position regarding the subsidy policy.

*Supportive associations:* These stakeholders are active in two forms: patient associations and charitable institutions. The role of NGOs such as Mahak is to facilitate and assist patients in paying healthcare costs. However, the role of patient associations such as the Haemophilia Association, Kidney Patients Association, and other similar associations is, first, is to defend the rights of their patients. The demanding role of associations is that they have held the government accountable to the citizens and are organized to claim their members' rights. Patient associations are highly sensitive to the types of services and quality of medicines because one of their members died due to hepatitis a few years ago. Secondly, their role is to reduce the economic burden on patients by negotiating with parliament members and the cabinet as they inform the Department of Special Diseases about new medicines or side effects of specific medications.

Supportive associations are in contact with patients with specific diseases and are essentially formed to serve these people, so they have a great interest in participating in the policy-making processes for these patients, including drug subsidies. Nonetheless, they do not have much organized official power to influence the policy-making process. According to interviewees P1 and P12, although associations do not have the official power to influence the policy of foreign currency subsidy for medicines, some supportive associations such as the Haemophilia Association and Thalassemia Association have been more active than other associations during the past decade and have shown their supportive position in the field of medicine through the media and in cases such as payment assistance for haemophilia patients. Interviewee R18 stated that patient associations have not been welcomed like before or have even been ignored by the government over the past few years because of NGOs and institutions such as the CFSD. Therefore, associations cannot influence the policy-making process for foreign currency subsidy allocation for medicines like other institutions such as the IFDA, because the government believes that the objectives of these associations run parallel with the NGOs' objectives.

In general, the government performance regarding involvement of NGOs in policy-making for specific patients has been weak, and patient associations have not contributed in making decisions because of negligence. In recent years, the establishment of the Deputy for Social Affairs within the MoHME, whose purpose is to encourage the participation of patient associations, has raised the level of engagement of such organizations in the decision-making process, including allocating foreign currency subsidies for medicines.

Interviewee P4 also discussed the role of associations:

*For example, until two years ago, few people knew EB, but now many people are aware of this disease. Journalists are not aware of diseases, but an NGO that knows how to approach an issue in the media increases interest. For example, Ilia was invited to the Khandavane TV Show, and they asked the charity organizations to help EB patients, yet few people knew about EB. The House of EB is an active group that is doing a big effort to increase EB's public awareness. For example, recently, a new agency and newspaper published several articles about EB. (P4)*

Some interviewees noted that some associations are more active than others and stated that due to problems related to infected blood, the MoHME has paid more attention to the associations related to these patients in recent years. Associations have sometimes increased public awareness about some unknown diseases and have drawn the attention of governmental actors and patients to such issues.

Regarding the role of associations, interviewee P1 noted:

*“Lobbyists are influential, particularly in handling special diseases; for example, haemophilia patients have a strong lobby. In the case of haemophilia, everything should be carefully examined before confirmation. But why don’t we consider the ‘burden of disease’? In Iran, the leading cause of death is cancer, followed by cardiovascular diseases and traffic accidents, but these groups don't have representatives, which means they have no effect.” (P1)*

Interviewee P13 believed that charitable institutions do not have an important role in medicine as NGOs. Considering the high price of medicines used by patients with genetic and chronic diseases in Iran, the government should engage these patients in decision-making. By increasing charities' involvement and pooling financial resources, the government can expand its supportive policies for medicines.

*Scientific associations:* The key role of scientific associations is to provide feedback to the Department of Special Diseases on the medicines consumed by patients with specific diseases. Suppose a new expensive drug is introduced to treat a special, incurable, or chronic disease. In that case, the relevant scientific associations will be asked to give their opinions regarding health insurance coverage and the indications for this medication. Scientific associations have little power or influence in drug subsidy decisions and are not interested in this issue.

*Media:* The media has a key role in decisions on subsidizing medicines. The media has a vital role in supporting or opposing a particular decision. For example, the media can promote supportive subsidies for patients as a national priority. In most cases, media coverage focuses on diseases and rarely interacts with the drug debate specifically. Supportive associations can promote subsidizing medicine through the media to stimulate general population's emotions and feelings. The media has contributed in drawing the attention of general population, policy-makers, and government towards several diseases. This issue also indicates the power of the media in promoting preventive activities through advertising and educational programmes, for example, the prevention of abortion in cases of rare and genetic fetal disorders. The power of the media can also be used for promoting the rational use of medicines.

*Media creates campaigns … which may be positive or negative … It can highlight any subject for the society.* (P12)

*There is no doubt about the impact of media, I mean, what social networks do. Suppose what medicines are subsidized? How much subsidies are allocating? Who decides and on which criteria? The media can make it transparent. The question is, currently our media keep saying why drug X is not widely available, and ask few questions about subsidies to medicines, while the media can play a prominent role.* (P5)

*CFSD:* As an NGO, the CFSD was established in 1996 with the collaboration of donors. The CFSD is the first organization supporting patients with specific diseases in the country. Its activities can be categorized into three broad categories: prevention, treatment, and education for special diseases (e.g., dialysis, thalassemia, and haemophilia) and severe illnesses (e.g., cancer, MS, kidney transplantation, diabetes, and EB). The mission of the foundation is to support patients with special and incurable diseases in all aspects, including medicines. Therefore, the CFSD also has a great interest in policies such as drug subsidies that increase patients' access to medicine. Thus, the CFSD has a supportive position, but it has limited power. It does update the media about shortage of medicine, and even sends informative letters to the Secretary-General of the UN about the impact of sanctions on access to medicine in Iran.

*Summary of findings of stakeholder analysis:* The stakeholder analysis for foreign currency subsidies for medicines included 21 stakeholders in five main categories: governmental organizations, NGOs, Iranian Parliament, general population, and the pharmaceutical industry. Research findings are based on the three dimensions of interest, power/influence, and position, as follows (Table 2):

*Interest:* Based on this dimension, the general population, Special Diseases Department in the MoHME, MCLSW, health insurance organizations, scientific associations, media, MIMT, pharmaceutical industry (distributors and exporters), and customs have low interest. The Iranian Parliament, MoHME, CBI, and pharmaceutical industry (manufacturers) have moderate interest. The government, PBO, IFDA, support associations, CFSD, and pharmaceutical industry (importers) have high interest.

*Power/influence:* Research findings based on the power dimension demonstrated that general population, the MCLSW, scientific associations, pharmaceutical industry (distributors and exporters), and customs have low power. The Department of Special Diseases of the MoHME, health insurance organizations, support associations, CFSD, MIMT, and the pharmaceutical industry (manufacturers and importers) have moderate power. The Iranian Parliament, Iranian Government, PBO, MoHME, IFDA, media, and CBI have high power.

*Position:* According to the position dimension, research findings demonstrated that the general population, the MCLSW, scientific associations, media, and the pharmaceutical industry (distributors and exporters) have a neutral position. The Department of Special Diseases of the MoHME, MIMT, and customs have low supportive position. Parliament, the IHIO, support associations, CFSD, CBI, and pharmaceutical industry (importers) have a moderate supportive position. In addition, the Iranian Government, MoHME, and IFDA have highly supportive positions. The pharmaceutical industry (manufacturers) has a position of opposition.

## Discussion

This study aimed to analyse the stakeholders involved in the allocation of foreign currency subsidies, a policy implemented by the Iranian Government targeting access to medicines. Stakeholder analysis helps in identifying the main stakeholders and evaluating their knowledge, interests, and interventional power [47, 58].

This study demonstrated that decision-making on cutting the foreign currency subsidies has a long history of debate that requires the participation of all stakeholders to reach consensus. Policies to improve access to medicines should be multisectoral and need to engage different stakeholders to achieve maximum support [59]. The study also revealed that an important challenge in allocating foreign currency subsidies is the dominance of a top-down approach in formulating and implementing policies, which affects the relationships between actors. According to the evidence, both top-down and bottom-up approaches should be employed to improve policies and their implementation, and to increase the continuity and success of policies [60]. Stakeholder participation is crucial to the success of the programmes, and therefore they should be involved from the outset (i.e., designing the project), as they will be influenced by the decisions and actions which will be taken. Their participation ensures that the legitimate interests and concerns of stakeholders are effectively addressed [61]. In particular, in allocating foreign currency subsidies for medicines in Iran, most decisions are made by the government, the MoHME, and the IFDA, generally excluding other stakeholders; establishing a dialogue could address the weaknesses of the policy.

Our findings showed that the identified stakeholders have a different range of interests, power/influence, and position in allocating foreign currency subsidies in Iran. Identification and analysis of stakeholders is essential for developing and implementing effective interventions and mobilizing all resources and facilities [62, 63]. In the case of allocating foreign currency subsidies in Iran, the identified stakeholders influence the formulation and implementation of policies, both directly and indirectly. Drawing on the participants' views, the government, the PBO, the MoHME, and the IFDA are among the main stakeholders. The findings revealed that the public sector, NGOs, and the private sector have little to moderate or high interest in the policy of allocating foreign currency subsidies. Different degrees of stakeholder interest may be associated with their different motivations [64]. Also, the public sector has medium or high power, and NGOs and the private sector have moderate or low power. Stakeholder power is one of the main components of developing and implementing health-related policies [65]. Despite the challenges in foreign currency allocation at a rate of 42,000 Iranian rial, most of the stakeholders have a supportive position, excluding domestic drug manufacturers.

This supportive position is due to the lack of an alternative policy option to ensure people's access to medicine, especially those with special and incurable or chronic diseases. Their concerns stem from the increased exchange rates in previous years and the subsequent decreased access to medicine. In the public sector, the MoHME and the IFDA have a high supportive position, while health insurance organizations—the IHIO and the SSO—hold a medium to high supportive position, and drug importers hold a moderate support position. In contrast, drug manufacturers were not supportive of the policy, and the distribution networks were neutral. The government is the most powerful institution regarding administrative affairs, as it can make decisions regarding the allocation of foreign currency subsidies. Given the political and social consequences of this decision, the required political will has not yet been established or institutionalized. The MoHME and the IFDA also have relatively high levels of power in this regard. One of the most substantial factors for these sectors was their scientific resources and information. Therefore, the MoHME and the IFDA can help the authorities produce scientific evidence that can be provided through policy discourse for formulating evidence-based policy. Some studies reported that recommendations for policy discourse to achieve interactive and inclusive policy-making have become widespread; if properly taken into account, such discourse will be participatory and evidence-based [66–69].

To mitigate the effects of economic crises, including that of the exchange rate, the Iranian Government has adopted a policy of allocating foreign exchange subsidies to medicines to increase society’s access to medicines. Several studies indicate that during economic crises, when people's access to medicine is threatened, different countries have adopted diverse policies to deal with it. Studies by Vandoros et al. [70] and Vogler et al. [71] on the European pharmaceutical system during economic crises show that governments have sought to increase patients' access to medicines in these countries by adopting policies such as reducing drug costs. A study conducted by Homedes and colleagues [72] revealed that Brazil and Argentina have improved patient access, which had been reduced by economic crises, by adopting policies such as reducing drug costs. Our study is consistent with these studies, which have emphasized the need for governmental intervention and policies to ensure the public’s access to medicines and to ensure health equity during economic crises.

In policy-making, no policy is permanent and fixed—in other words, it should be flexible and able to be revised in the short and long run. According to the findings, it seems that the government's supportive policies, such as allocating foreign currency subsidies, require revision; however, a detailed analysis of consequences is necessary. Afterwards, the following measures can be used to delineate the foreign currency subsidy for medicines:

1. *Inflation due to reduced subsidies:* When the foreign currency subsidy for medicines is eliminated, the government should pay the released funds (in rials) to health insurance organizations, which will be a considerable amount as the market exchange rate has soared in recent years. On the other hand, the budget shortage is high; therefore, this intervention will aggravate this shortage and result in higher inflation. Thus, finding a sustainable source of financing is required before any intervention.
2. *Distrust of the government regarding allocation of the released funds for medicines:* Mutual trust between the government and the general population is one of the main pillars of society. Good governance is associated with higher levels of trust in governments. In recent years, various events, such as the hike in gasoline prices and its consequences, and ineffective management of the recent natural disasters, have widened the gap between the citizens and the government. Accordingly, this distrust has engendered suspicion among general population about the government’s intention to pay the equivalent rial subsidy, which might adversely affect people's access to medicines. Hence, the necessary plans must be developed to ensure timely allocation of rial subsidies through sustainable findings by emphasizing high-level documents.
3. *The possibility of corruption:* If foreign currency subsidy is cut, the possibility of informal payments by importers and producers of medicines to receive a subsidized exchange rate would be high. Therefore, it is necessary to anticipate measures against possible corruption.
4. *The possibility of jeopardizing access to medicine, especially for uninsured populations:* The country is in the early stages of universal health insurance, and some low socioeconomic groups, despite various protective laws, are uninsured. Hence, ensuring access to medicines in the absence of foreign currency subsidy is essential. Also, special attention should be paid to migrants and those without a national identity. Therefore, planning for basic insurance coverage is essential to ensure access to medicine.
5. *The possibility of replacing over-the-counter (OTC) medicines with similar medicines covered by insurance:* Elimination of foreign currency subsidies may result in increased prices of OTC medications. On the other hand, it is expected that physicians will be asked to prescribe similar medicines covered by insurance. Hence, the consumption of alternative OTC medicines with insurance coverage will increase, particularly those with lower prices, even after paying the copayment. Importantly, special attention should be paid to the moral hazard.
6. *The possibility of speculation after removing subsidies:* Even fake news about removing the exchange rate of 42,000 Iranian rial for essential goods, including medicine, can trigger a wave of price increase, which may also cause monopoly or oligopoly of medicines by some importers, manufacturers, and distribution networks, which in turn may reduce the access to medicines. Thus, special attention should be paid to preventing the negative implications.

The authors recommend that, after analysing the consequences of reducing or eliminating the foreign currency subsidy, the following interventions need to be implemented for gradual elimination of such subsidies:

1. The gradual removal of subsidies: In the first year, foreign currency subsidies for OTC medicines, some low-consumption medicines for acute diseases, and imported medicines should be removed. Next, in the second year, based on the first year's experiences and by evaluating the policy gaps, the same policy should be applied to domestic products.
2. Adjusting the price of medicines simultaneously with subsidy removal.
3. Revising the Medical, Pharmaceutical, Food, and Beverage Regulations Act (1955) (amended in 1988) to transfer the commercial or trade activities of the IFDA to the MIMT, with pricing delegated to syndicates.
4. The development of national eHealth architecture in health system [73, 74] and establishment of the essential infrastructure in health insurance organizations to adopt and implement eHealth solutions like electronic health records (EHR).
5. Obliging the government, through high-level documents, to pay the released funds resulting from reducing subsidies to health insurance organizations.
6. Earmarking the funds released through these resources to ensure that they are not spent for other purposes in insurance organizations.

This study has a number of strengths and limitations. We used the approach developed by Varvasovszky and Brugha to analyse the stakeholders of the policy of allocating foreign currency subsidy to medicines in Iran [45]. To better understand the policy structure, the authors recommend social network analysis (SNA). Using this approach would help create constructive conversation and interaction with stakeholders based on their position, interest, and influence [75]. In this study, we collected the data through semi-structured face-to-face interviews, which are regarded as superior for the generation of rich narrative data [76]; however, for enriching the results with more information, the authors recommend using focus group discussions in further studies. Some of the participants were among the policy stakeholders; they probably did not declare their real position regarding the policy. Another limitation was the difficulty in reaching some interviewees and the long waiting time to conduct the interviews. Also, we tried to interview all stakeholders, but we could not access all of them. Another limitation is the fact that individuals may change their views over time. The opinions of the interviewed representatives of each organization might not be generalizable to the entire organization.

Finally, this research is nonrandom in nature in selecting interviewees due to the snowball sampling method. The dominant characteristics of the snowball sampling are dependent on selection bias. In this sampling, selection of interviewees was based on the subjective judgements of key informants and may therefore be influenced by numerous considerations not easily assessed or controlled by researchers [77, 78]. However, considering the use of a mixed sampling method in this study, we tried to somewhat overcome this limitation. Despite these limitations, this stakeholder analysis is an appropriate tool for evidence-informed policy-making.

## Conclusions

Medicines and medical supplies have been always critical for healthcare services provision, and with a substantial role in ensuring and enhancing society health. Accordingly, access to medicines globally utilised for assessing the performance of health systems. Governments usually take various measures to create a reliable access via protecting the eligible against catastrophic health expenditures. This study demonstrated that identifying stakeholders and challenges related to the policy of allocating foreign currency subsidies might help policy-makers gain a comprehensive understanding for workable decision-making. Stakeholder analysis is de facto a starting point for developing a policy by gathering stakeholders’ concerns and preferences. The results also indicated that an effective allocation of foreign currency subsidy requires active participation of several stakeholders, including the PBO, MoHME, and the IFDA, with a highly supportive position. Although, the domestic manufacturers expressed an opposing position. The authors recommend a gradual reduction in foreign currency subsidy only if all the requirements are met. Revision of the current policy along with the abovementioned requirements (which is crucial to strengthen achieving UHC by reducing OOP and inequality in access to medicines), in addition to financial transparency and improved efficiency, might also pave the way towards achieving the SDGs by improving access to medicines. Other countries with similar situations are advised to consult our results for initiating their interventions regarding access to medicines.

## Supplementary Information

**Additional file 1.** Interview guide.

## Data Availability

The data gathered during this study are not publicly available as consent was not obtained for such sharing.

## Abbreviations

MoHME: Ministry of Health and Medical Education
IFDA: Iran Food and Drug Administration
MDGs: Millennium Development Goals
UN: United Nations
LMICs: Low- and middle-income countries
SDGs: Sustainable Development Goals
UHC: Universal health coverage
OOP: Out-of-pocket
THEs: Total health expenditures
GDP: Gross domestic product
NDP: National drug policy
CBI: Central Bank of Iran
PBO: Planning and Budget Organization
BHIBPs: Basic health insurance benefit packages
MS: Multiple sclerosis
NDPs: National development plans
NGOs: Nongovernmental organizations
DCA: Directed content analysis
TUMS: Tehran University of Medical Sciences
MCLSW: Ministry of Cooperatives, Labour, and Social Welfare
MIMT: Ministry of Industry, Mining, and Trade
SSO: Social Security Organization
IHIO: Iran Health Insurance Organization
AFMSIO: Armed Forces Medical Services Insurance Organization
IKRF: Imam Khomeini Relief Foundation
CFSD: Charity Foundation for Special Diseases
OTC: Over-the-counter
SNA: Social network analysis
