# Supplementary material for: Pharmaceutical subsidy policy in Iran: a qualitative stakeholder analysis
Source: Health Res Policy Syst. 2021 Dec 23;19:150. doi: 10.1186/s12961-021-00762-6 (PMC8697451; doi:10.1186/s12961-021-00762-6)
Supplement: Supplementary file 1 — Additional file 1. Interview guide. [file 12961_2021_762_MOESM1_ESM.docx]

Additional File 1

**Interview guide**

Interviewee code:

Position:

Work address:

Age:

Gender:

Work experience:

Interview Date:

1. In your opinion, what definition is appropriate for expensive medicines in the country, and what boundary do you accept for the identification of expensive medicines?
2. What policies and programs on financial protection are available in the country for increasing access of patients who suffer from special and incurable diseases? Can you mention their strengths and weaknesses?
3. Why a financial protection program has been developed for patients who suffer from special diseases? How do you evaluate this program? What is the difference between this program and other supportive programs and measures?
4. Who are the actors (i.e. individuals, organizations, and government) and the influential stakeholders of the medicine subsidy allocation policy?
5. What is their position, power, interest, and communication in this process?
6. Do all stakeholders, including people (community), patients, associations, and healthcare providers, are involved in the process of making decisions on allocating subsidies to medicines? If yes, how? If no, what is the reason for their non-participation? Does the media (national broadcasting, newspapers, and virtual networks) has an effective influence over the process of allocation of medicine subsidies?
7. Does the policy of allocating subsidies to medicines (real details and structural components, comprehensiveness, and completeness in terms of considering drugs related to rare, specific, incurable, and chronic diseases, infertility, etc. as well as the amount and percentage of each drug) has an appropriate configuration? What are its strengths and weaknesses?
8. What are the challenges related to allocating subsidies to medicines in the country? What solutions do you recommend to achieve the desired situation?
